# A New Muscarine-Containing *Inosperma* (Inocybaceae, Agaricales) Species Discovered From One Poisoning Incident Occurring in Tropical China

**DOI:** 10.3389/fmicb.2022.923435

**Published:** 2022-07-04

**Authors:** Lun-Sha Deng, Wen-Jie Yu, Nian-Kai Zeng, Yi-Zhe Zhang, Xiao-Peng Wu, Hai-Jiao Li, Fei Xu, Yu-Guang Fan

**Affiliations:** ^1^Key Laboratory of Tropical Translational Medicine of Ministry of Education, Hainan Key Laboratory for R&D of Tropical Herbs, Tropical Environment and Health Laboratory, School of Pharmacy & International School of Public Health and One Health, Hainan Medical University, Haikou, China; ^2^National Institute of Occupational Health and Poison Control, Chinese Centre for Disease Control and Prevention, Beijing, China; ^3^Ningxia Key Laboratory of Environmental Factors and Chronic Diseases Control, School of Public Health and Management, Ningxia Medical University, Yinchuan, China; ^4^Analysis and Test Center, Chinese Academy of Tropical Agricultural Sciences, Haikou, China

**Keywords:** new species, mushroom poisoning incident, muscarine, ultra-high liquid chromatography triple quadrupole mass spectrometry, tropical China

## Abstract

Currently, mushroom poisoning still poses a huge problem to humans' health and life globally. Poisoning incidents caused by *Inosperma* spp. were reported continuously in tropical China in recent years. In this study, a new poisonous *Inosperma* species, discovered from a poisoning incident, was described in tropical China based on morphological, molecular, and toxin detection evidence; detailed descriptions, photographs, and comparisons to closely related species were provided. For qualitative analysis, through targeted screening using ultra-high liquid chromatography triple quadrupole mass spectrometry (UPLC-MS/MS), the new species contains muscarine and no other toxins (two isoxazole derivatives, two tryptamine alkaloids, three amatoxins, and three phallotoxins). For quantitative analysis, muscarine contents in the pileus and the stipe were 2.08 ± 0.05 and 6.53 ± 1.88 g/kg, respectively.

## Introduction

Mushroom poisoning is one of the leading causes of foodborne outbreaks and outbreak-associated deaths in China (Li et al., 2020). The latest study reported 923 patients and 20 deaths caused by 74 different poisonous mushrooms, which were involved in 327 investigations in 2021 in China (Li et al., 2022). Currently, over 500 toxic mushroom species were reported in China. However, undocumented mushroom species were constantly discovered in different mushroom poisoning incidents (Li et al., 2020, 2021a, 2022). Among these diverse groups of poisonous mushrooms, Inocybaceae is supposed to contain a large number of muscarine-containing taxa. The species diversity of Inocybaceae was documented by a series of studies in China (Fan and Bau, 2010, 2013, 2014a,b, 2016, 2017, 2018, 2020; Bau and Fan, 2018; Fan et al., 2018; Yu et al., 2020; Deng et al., 2021a,b; Li et al., 2021b). *Inosperma* genus only includes a few toxic species, but a high level of muscarine was frequently detected (Kosentka et al., 2013; Sailatha et al., 2014; Latha et al., 2020; Deng et al., 2021a; Patocka et al., 2021) and was reported constantly in mushroom poisoning incidents from tropical Asia (Chandrasekharan et al., 2020; Parnmen et al., 2021; Li et al., 2022).

*Inosperma* (Kühner) Matheny & Esteve-Rav. belongs to Inocybaceae Jülich. At present, 77 taxa were recorded in the Index Fungorum database (www. indexfungorum.org; retrieved April 11, 2022). The genus is characterized by rimose or scaly pileus, often reddening context, elliptic basidiospores, and thin-walled cheilocystidia. Muscarine, a neurotoxin found in certain *Inosperma* taxa, has clinical manifestations of this type of poisoning substance that is often associated with the rapid onset (15 min−2 h) of classic parasympathetic stimulation, the triad of which increased sweating, salivation, and lachrymation. Other symptoms may include pupil constriction, blurred vision, urgent or painful micturition, nasal discharge or congestion, asthma/bronchoconstriction, hypotension, bradycardia, skin flushing, watery diarrhea, vomiting, and abdominal pain/colic. In fact, the more rapid the onset, the more severe the intoxication. Severe poisoning is potentially lethal, though death is a rare outcome and unlikely if appropriately treated with atropine (Lurie et al., 2009; White et al., 2019). The latest toxic metabolite profiling analysis and the toxicokinetic study confirmed that muscarine was the main toxic substance in *I. virosum* (KB Vrinda, CK Pradeep, AV Joseph, and TK Abraham ex CK Pradeep, KB Vrinda and Matheny) Matheny & Esteve-Rav (Latha et al., 2020).

On May 9, 2020, 10 people aged 20–94 from two families were poisoned by an undescribed *Inosperma* species in Wanning, Hainan Province, China. They showed classic parasympathetic stimulation syndromes, including sweating, salivation, and lachrymation; other symptoms included blurred vision, nausea, vomiting, abdominal pain, and tachycardia. All patients recovered with supportive treatments within 24 h. Limited materials of the poisonous mushroom were obtained from this poisoning locale in 2020. Fortunately, in 2021, this species was encountered again in Wuzhishan, Hainan with plentiful individuals. Accordingly, the new species is described with morphological characters and phylogenetic analyses in the present study. For better understanding of its toxin type and contents, a targeted screening of toxin and quantitative analysis were performed using a comprehensive method of UPLC-MS/MS.

## Materials and Methods

### Field Sampling and Morphological Studies

Poisoned mushroom specimens responsible for the poisoning were immediately collected from the locality where the victims had cooked and ingested them in Shanjia Village, Changfeng Town, Wanning City (Hainan, China) on 9 May 2020. The holotype specimen was collected under *Castanopsis hainanensis* Merr. (Fagaceae) mixed with *Liquidambar formosana* Hance (Hamamelidaceae), *Microcos paniculata* Linn. (Tiliaceae), *Psychotria rubra* (Rubiaceae), *Machilus chinensis* (Champ. ex Benth.) Hemsl. (Lauraceae), and *Garcinia oblongifolia* Champ. ex Benth. (Clusiaceae) on 11 August 2021 from Wuzhishan, Hainan Province in tropical China. Fresh specimens were photographed *in situ*, and the descriptions were done as soon as possible after fieldwork with color codes, following the study of Kornerup and Wanscher (1978). Collected samples were dried at 45°C with an electronic drier overnight and sealed in plastic bags with silica gels for long-term preservation (Yu et al., 2020). Dried specimens were deposited in the Herbarium of Changbai Mountain Natural Reserve (ANTU) with FCAS numbers and the Fungal Herbarium of Hainan Medical University (FHMU). The methods of morphological study similar to that in the study of Deng et al. (2021a) with some modifications. Microscopic structures were observed from dried specimens mounted in KOH (5%) or stained with Congo red solution (1%) when necessary. At least 20 basidiospores were randomly selected for each individual, and the apiculus was excluded when measured. Numbers in square brackets [a/b/c] mean “a” basidiospores measured from “b” basidiomata of “c” individuals. Additionally, the dimensions results of basidiospores (length × width) are shown as (d) e–**f**–g (h) × (i) j–**k**–l (m), where “d” is the minimum length, “e–g” means a minimum of 90% of the measured values, “h” is the maximum of value (“d” < 5^th^ percentile; “h” > 95^th^ percentile) and “f” represents the average length of total measured basidiospores and width “i–l,” “k,” and “i–m” is shown in the same format. Q is the ratio of length/width for each basidiospore, the average of Q is shown as Q_m_, and Q_m_ ± SD represents Q_m_ ± the sample standard deviation (Ge et al., 2021).

### DNA Extraction, PCR Amplification, and Phylogenetic Analyses

Genomic DNA was extracted from dried specimens using the NuClean Plant Genomic DNA kit (ComWin Biotech, Beijing) for rapid DNA extraction and amplification. The ITS, nrLSU, and RPB2 gene regions were amplified using the primer pairs of ITS1F/ITS4 for ITS (Gardes and Bruns, 1993), LR0R/LR7 for nrLSU (Vilgalys and Hester, 1990), and bRPB2-6F/bRPB2-7.1R for RPB2 (Matheny, 2005). The settings of PCR cycles were based on the study of Wang et al. (2021). Then, the PCR products were sent to the Beijing Genomics Institute for purification and sequencing.

The sequences (ITS, nrLSU, and RPB2) obtained in this study were verified (Nilsson et al., 2012) and deposited in GenBank (Supplementary Table S1 in Supplementary Material 1). Sequences of *Inosperma* taxa mainly retrieved from previous studies (Larsson et al., 2009; Kropp et al., 2013; Horak et al., 2015; Naseer et al., 2017; Bau and Fan, 2018; Matheny and Kudzma, 2019; Matheny et al., 2020; Aïgnon et al., 2021; Bandini et al., 2021; Cervini et al., 2021; Deng et al., 2021a,b) were downloaded from GenBank for phylogenetic analyses (Supplementary Table S1 in Supplementary Material 1). MAFFT online service was used to align each of the three genes with the E-INS-i iterative refinement strategy (https://mafft.cbrc.jp/alignment/server/); then the alignments were manually adjusted by BioEdit version 7.0.9.0 (Hall, 1999). The best-fit evolutionary model for each gene partition was selected by MrModeltest v2.3 with the AIC criterion (Nylander, 2004). The dataset of the individual locus was concatenated using MEGA 5.02 (Tamura et al., 2011). *Auritella hispida* Matheny & T.W. Henkel and *A. spiculosa* Matheny & T.W. Henkel were served as outgroups (Matheny et al., 2020). Maximum likelihood (ML) analyses were performed in the W-IQ-TREE web service (http://iqtree.cibiv.univie.ac.at/) with 1,000 ultrafast bootstrap replicates (Trifinopoulos et al., 2016). Bayesian inference (BI) analyses were carried out in MrBayes v.3.2.7a (Ronquist et al., 2012) with the selected model for each partition. Four Markov chains (MCMCs) were set to run for 100 million generations and automatically terminated using the stoprul and stopval commands when the standard deviation of the split frequencies fell below.01, with sampling for every 100th generation. The first 25% of trees were discarded (Ronquist et al., 2012).

### Sample Pretreatment

Dried mushroom samples were separated into two parts: the pileus and the stipe. A 20 mg powdery sample of the pileus and the stipe was weighted into different tubes, respectively; then, 2 mL of methanol-water (5:95, v/v) was added, and the mixture was treated in an ultrasonic bath for another 30 min after being vortexed for 30 min; next, the mixture was centrifuged at 1,000 rpm for 5 min. Afterward, the total supernatant was collected using a 0.22 μm organic filter membrane to filtrate before UPLC-MS/MS analysis and diluted with acetonitrile water (7:3, v/v) when necessary. *Lentinula edodes* (Berk.) Pegler was used as the blank sample. The analytical results are reported as mean±SD g/kg, where mean is the average content of muscarine and SD represents standard deviation.

### UPLC-MS/MS Parameters

The standards of muscarine, two isoxazole derivatives (ibotenic acid, muscimol), two tryptamine alkaloids (psilocybin, psilocin), three amatoxins (α-amanitin, β-amanitin, and γ-amanitin), and three phallotoxins (phalloidin, phallacidin, and phallisacin) in methanol were purchased from the Alta Scientific Co., Ltd. (Tianjin, China) as certified reference materials.

The mushroom toxin detection used the US Waters Co., LTD. ultrahigh performance liquid chromatography-tandem mass spectrometry (UPLC-MS/MS). For detailed sample preparations and the analysis of muscarine, amatoxins, and phallotoxins, we referred to the study of Xu et al. (2020a,b).

Features of the UPLC-MS/MS detection of ibotenic acid and muscimol were as follows. The chromatographic separation was conducted using an ACQUITY UPLC C8 column (2.1 × 100 mm, 1.7 μm; Waters, USA). The mobile phase consisting of acetonitrile (A) and 4% formic acid aqueous solution (B) at a flow rate of 0.3 mL/min was used for elution within 5 min as follows: 0 → 1 min, 2% A; 1 → 2 min, 2 → 70% A; 2 → 3 min, 70% A; 3 → 3.5 min, 70 → 2% A; and 3.5 → 5 min, 2% A. The analytical column was kept at 40°C. A volume of 10 μL sample extraction was injected into the instrument. The positive MS/MS conditions can refer to muscarine (Xu et al., 2020a). The ion pairs were 115.1 > 68.1 (Cone at 16 V; Collision at 12 V) and 159.1 > 113.1 (Cone at 16 V; Collision at 12 V) for ibotenic acid as well as 115.1 > 98.1 (Cone at 15 V; Collision at 10 V) and 115.1 > 68.1 (Cone at 15 V; Collision at 18 V) for muscimol.

To determine the presence of psilocybin and psilocin, the ACQUITY UPLC T3 column (2.1 × 100 mm, 1.7 μm; Waters, USA) was used as a separation column. The mobile phases were acetonitrile (A) and 10 mmol/L ammonium acetate aqueous solution (B). The gradient elution was set as follows: 0 → 0.5 min, 0%A; 0.5 → 4 min, 0 → 85%A; 4 → 4.5 min, 85%A; 4.5 → 5 min, 85 → 0%A; and 5 → 7 min, 0%A. The whole running time was 7 min with the column temperature at 40°C and the injection volume of 10 μL. We considered other instrument parameters as reported previously by Xu et al. (2020a). The psilocybin ion pairs were 285.1 > 85.2 (Cone at 16 V; Collision at 18 V), 285.1 > 240.1 (Cone at 16 V; Collision at 17 V), 205.1 > 58.2 (Cone at 26 V; Collision at 13 V), and 205.1 > 160.1 (Cone at 26 V; Collision at 13 V).

## Results

### Phylogenetic Analyses

**Figure 1:**
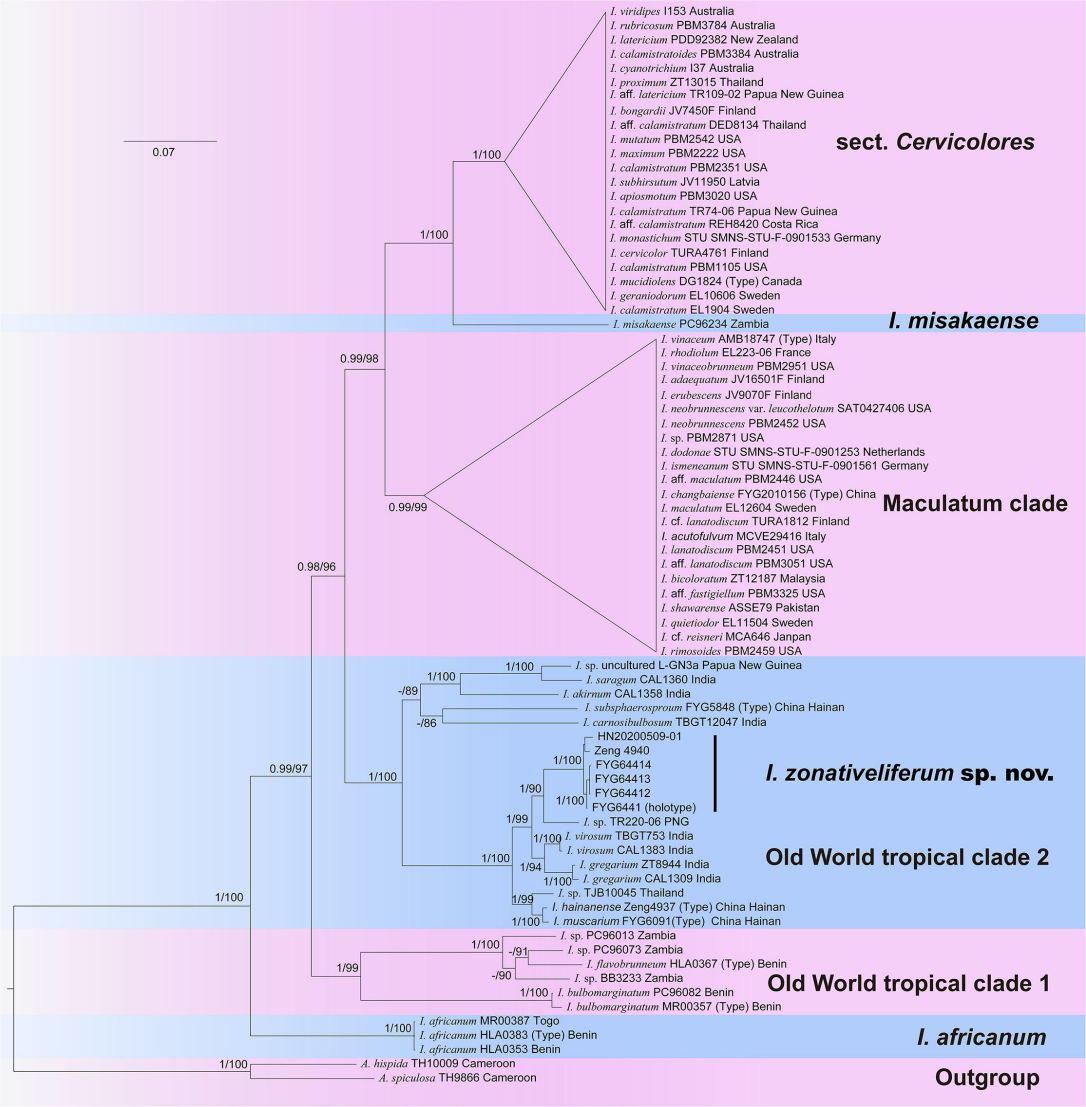
Phylogram generated by Bayesian Inference (BI) analyses based on sequences of a combined dataset from nuclear genes (rDNA-ITS, nrLSU, and RPB2), rooted with *Auritella hispida* and *A. spiculosa*. Bayesian inference posterior probabilities (BI-PP) of ≥0.95 and ML bootstrap proportions (ML-BP) of ≥70 are represented as BI-PP/ML-BP. *Inosperma zonativeliferum* is the newly described taxa.

The final multilocus dataset includes 76 taxa and 15 new sequences (6 ITS, 5 LSU, and 4 RPB2) of the new species generated in the present study. The concatenated dataset comprises 3,163 nucleotide sites with 823 bp ITS, 1,560 bp LSU, and 780 bp RPB2, of which 1,827 are constant and 962 are parsimony informative (Supplementary Data Sheet 3, alignment data sheet for phylogenetic analysis). The best-fit evolutionary models of three genes are all the same as the GTR+I+G model. The best-scoring trees of maximum likelihood (ML) and Bayesian analyses are presented in Figure 1. Six major clades were revealed by the three-gene phylogeny, namely sect. *Cervicolores, I. misakaense* lineage, Maculatum clade, Old World tropical clade 1, Old World tropical clade 2, and the *I. africanum* lineage. All specimens of the new species were grouped in an independent lineage with full supports (BP = 100%, PP = 1) nested in the Old-World tropical clade 2 and was sister to the lineage unifying *I. virosum I. gregarium* (K.P.D. Latha & Manim.) Matheny & Esteve-Rav., and an undescribed species from Papua New Guinea (*Inosperma* sp. TR220-06; BP = 90%, PP = 1).

### Taxonomy

**Figure 2:**
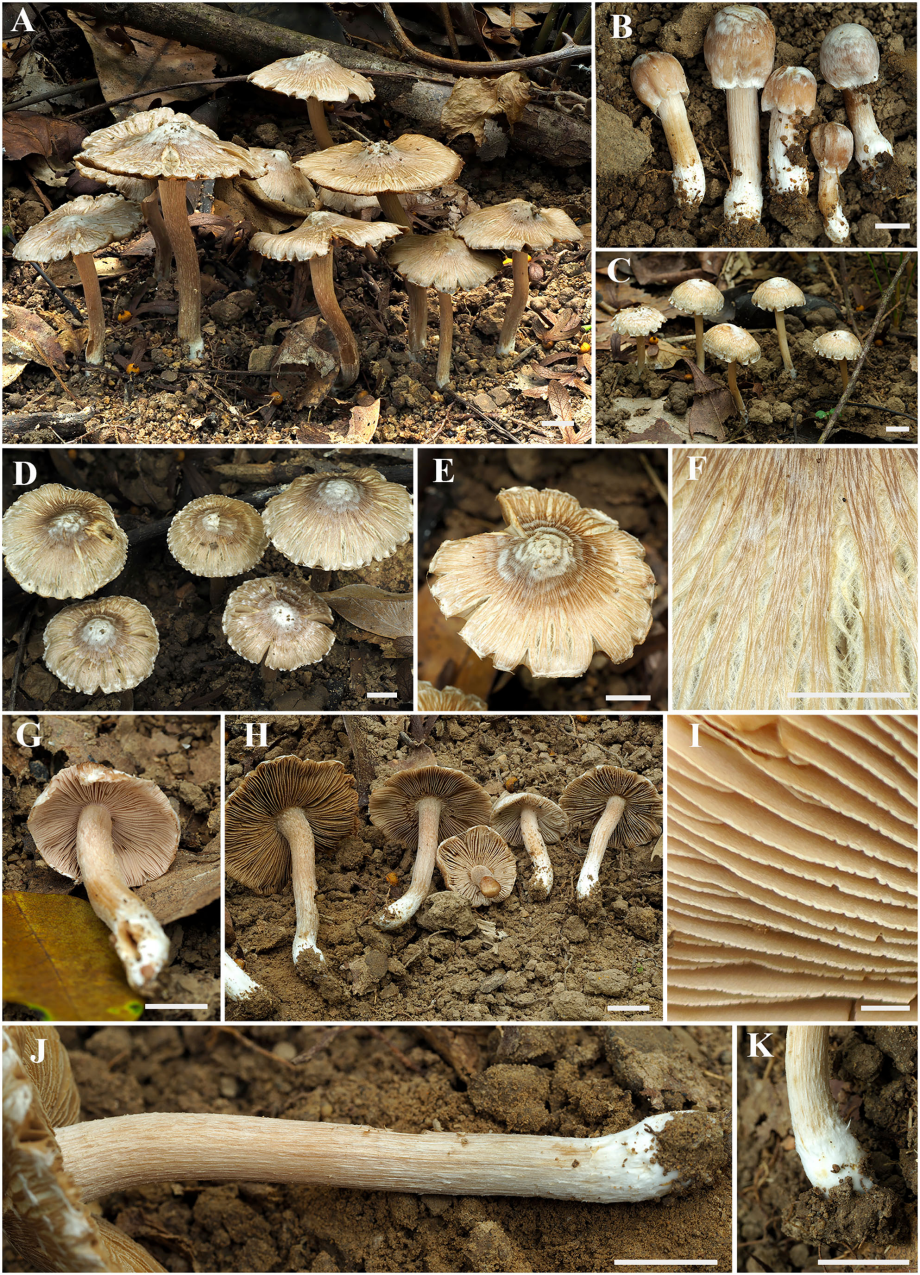
Basidiomata of *Inosperma zonativeliferum*. **(A–D)** Basidiomata; **(E,F)** Rimose to rimulose pileus; **(G,H)** Lamellae; **(I)** Lamellae edge; **(J,K)** Stipe surface. Scale bars: **(A–H, J,K)** = 10 mm, **(I)** = 1 mm. Photos by Y.-G. Fan.

**Figure 3:**
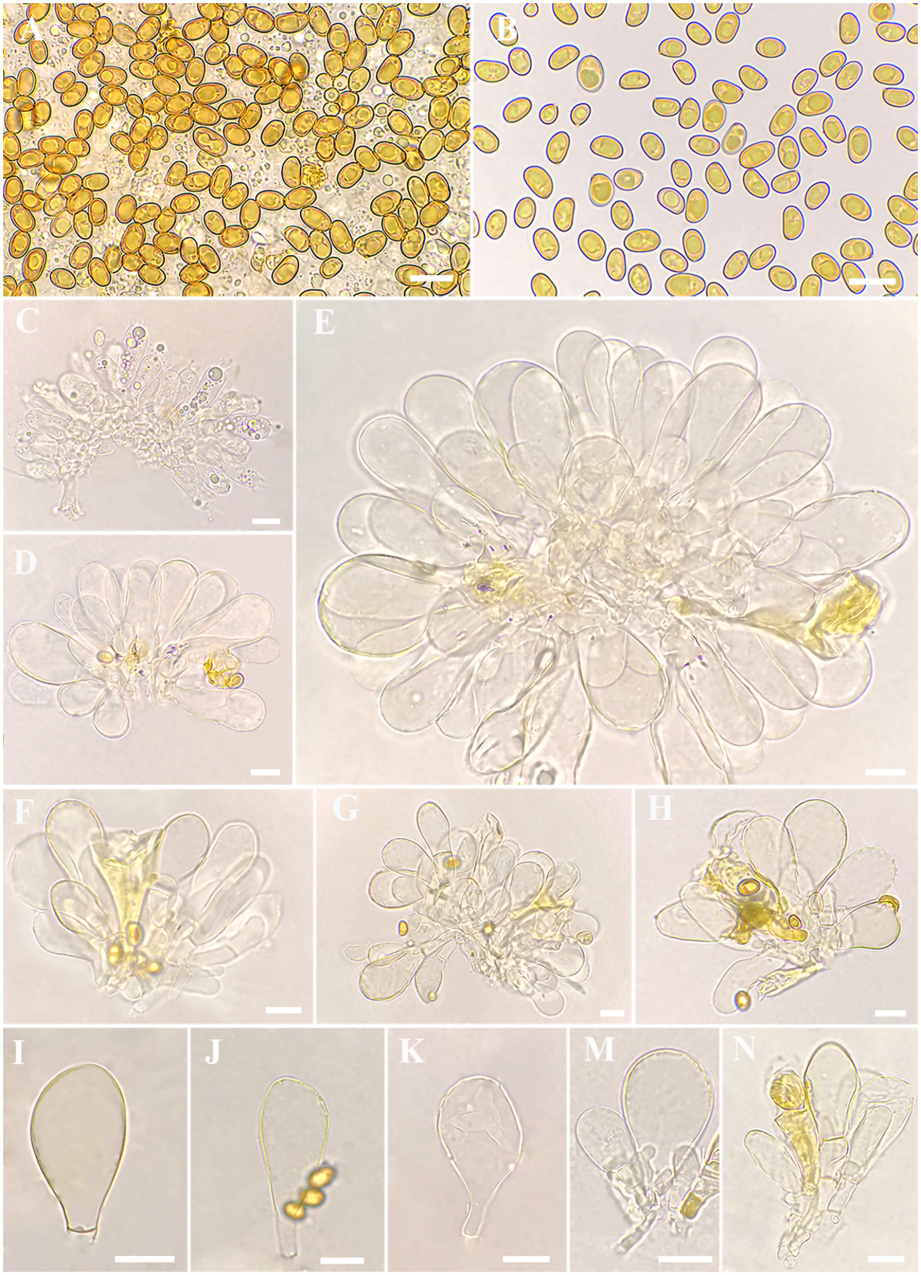
Microscopic features of *Inosperma zonativeliferum* (FCAS3509, holotype). **(A,B)** Basidiospores; **(C)** Basidia; **(D–H)** Cheilocystidia in clusters; **(I–N)** Cheilocystidia. Scale bars: **(A–N)** = 10 μm. Photos by L.-S. Deng.

**Figure 4:**
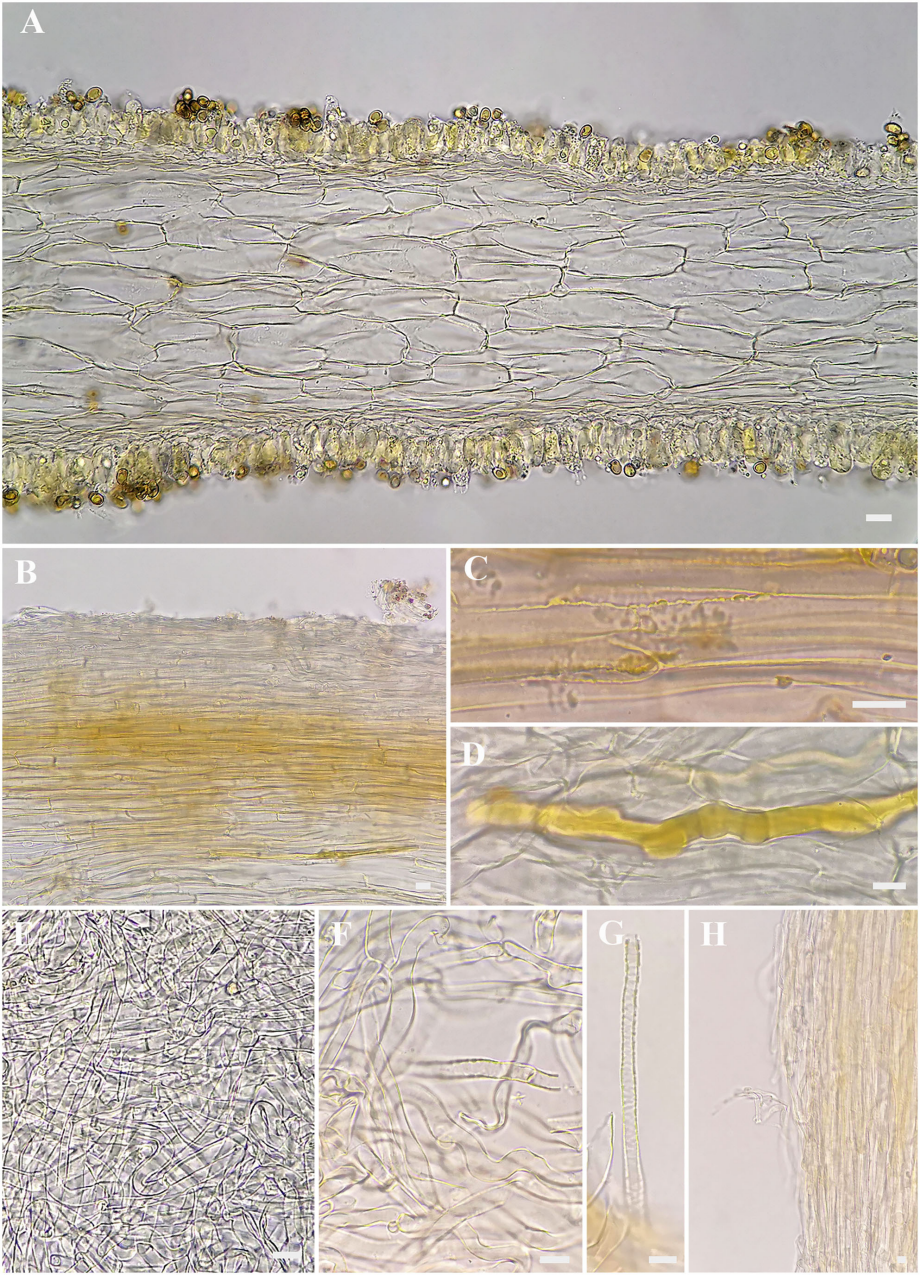
Microscopic features of *Inosperma zonativeliferum* (FCAS3509, holotype). **(A)** Hymenophoral trama; **(B,C)** Pileipellis and pileal trama; **(D)** Oleiferous hyphae; **(E–G)** Veilpellis; **(H)** Stipitipellis and stipe trama. Scale bars: **(A–H)** = 10 μm. Photos by L.-S. Deng.

**Figure 5:**
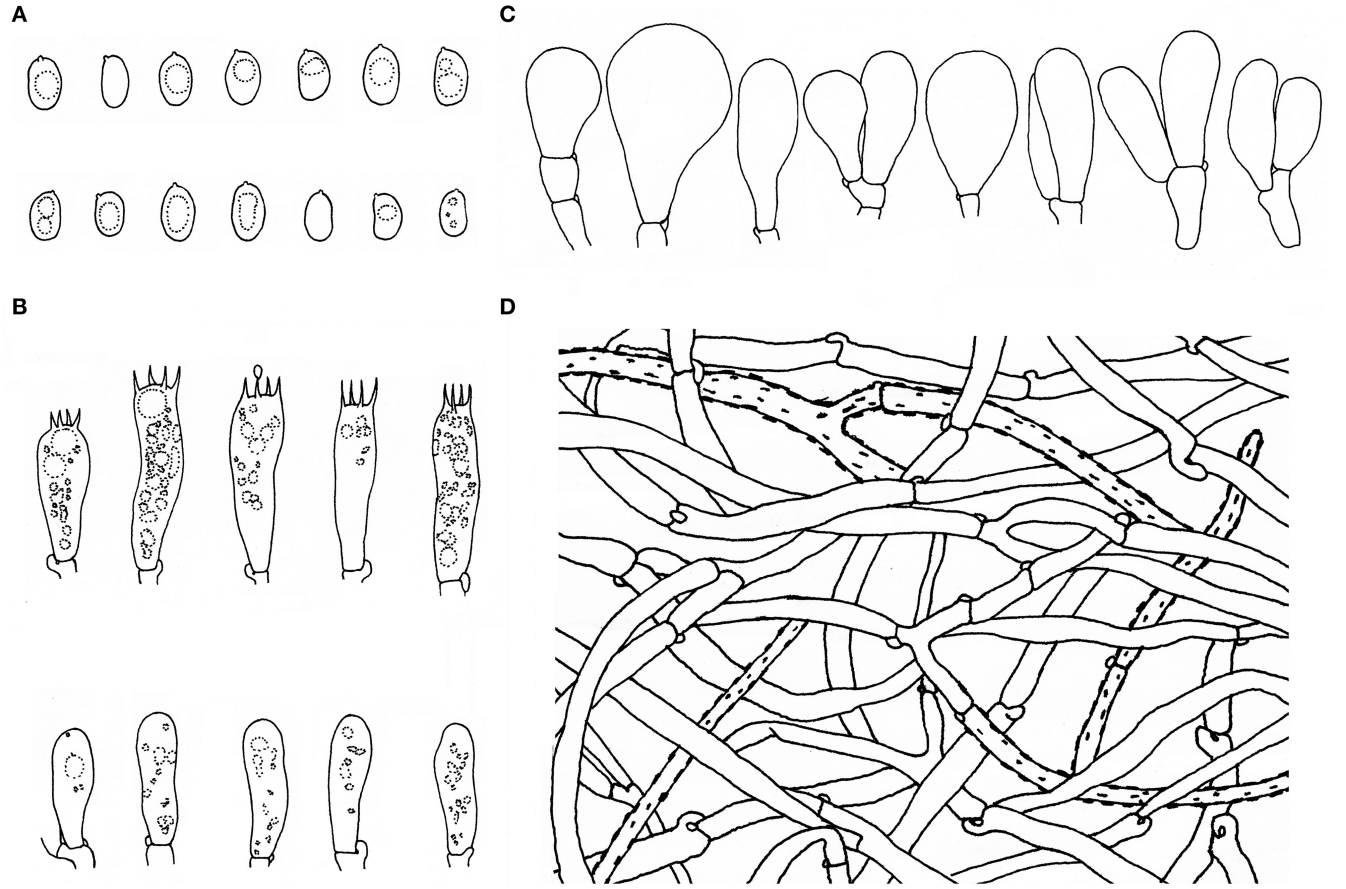
Microscopic features of *Inosperma zonativeliferum* (FCAS3509, holotype). **(A)** Basidiospores; **(B)** Basidia and basidioles; **(C)** Cheilocystidia; **(D)** Veilpellis. Scale bars: **(A–D)** = 10 μm. Drawings by H.-J. Li.

***Inosperma zonativeliferum*** Y.G. Fan, H.J. Li, F. Xu, L.S. Deng & W.J. Yu sp. nov. (Figures 2–5).

Mycobank: MB 843696

***Inosperma zonativeliferum*** is characterized by its medium-sized basidiomata, often a split pileus with thick white to dirty white veil remnants, pinkish or slightly orange-tinged lamellae, and a stipe with a distinct white base. Ellipsoid basidiospores and two types of thin- to slightly thick-walled cheilocystidia are present. It differs from other taxa in the Old World tropical clade 2 by its yellowish pileus with thick, persistent, and zonate velar remnant.

*Type*—**CHINA**. Hainan Province, Wuzhishan, Maoyang Town, Maoyang Village, 11 August 2021, FYG6441 (Holotype, FCAS3509), GenBank accession numbers: ITS (OL850878), nrLSU (OM845772), RPB2 (ON075044).

*Etymology*. —*Zonativeliferum*, refers to its thick, persistent, and zonate velar remnants on the pileus.

Basidiomata small to medium-sized. Pileus 8–69 mm in diameter, conical convex when very young, becoming broadly convex to plano-convex with age, with an indistinct umbo in young and middle age, then broadly umbonate when over-mature; margin initially incurved, slightly decurved with age; crenate, becoming strongly reflexed when old; surface dry, smooth, never or rarely split when young, becoming radially fibrillose-rimulose, splited to strongly rimose when old, covered with distinct, thick ivory white veil remnants all over the pileus in very young age, then becoming radially appressed and scaly with ivory white to dirty white (1A2) veil remnants, pale chocolate (6B3) to dark brown (6C4) around the disc, fibrils faint yellowish brown (4A3), background yellowish white (4A2) or grayish white (3B1) to brownish gray (4B2). Lamellae rather crowded, adnexed, initially pale pinkish (7A2) to yellowish white (5A3), becoming yellowish brown (5B4) to dark brown (5C4) with orange tinge (5B7) when old, 1–3.5 mm wide, often with unequal lamella, edge fimbriate, slightly serrate to wavy when matured. Stipe 20–95 × 5–6 mm, central, solid, terete, often slightly swollen at the apex and base, furfuraceous with some white squamulose at the stipe apex, longitudinally fibrillose downward the stipe; base with distinct ivory white, tomentose hyphae; surface white to yellowish white (5A3), becoming yellowish brown (5B5) when touched. Context solid, fleshy in pileus, 0.35–1.3 mm thick at mid-radius, up to 1.7 (2) mm thick under the umbo, uniformly white to slightly yellowish white (4A2), slightly yellowish white (4A2) or pale pinkish white (5A2) at first, becoming yellowish brown (4B3) to olive green (4C4) when old or cut; base often hygrophanous when being cut, fibrillose and striate in the stipe. Odor fungoid or grassy.

Basidiospores [500/10/6] 8–**8.8**−9.9 (11.1) × 5–**5.6**−6.3 (7) μm, Q = (1.27) 1.40–**1.57**−1.75 (1.90), Q_m_ ± SD = 1.57 ± 0.123, mostly ellipsoid, sometimes phaseoliform to sub-phaseoliform, obovoid, occasionally sub-globose, smooth, slightly thick-walled, often with an indistinct apiculus, usually with a round to amorphous yellowish brown oily contents. Basidia 21–31 × 8–11 μm, mostly clavate, sometimes broadly clavate, obtuse at apex, then somewhat tapered toward the base, 4-spored, sterigmata up to 6 μm in length, thin-walled, hyaline to pale yellow, often with faint yellow oily droplets, sometimes with golden yellow to yellowish brown contents. Pleurocystidia none. Lamella edge sterile. Cheilocystidia of two types: (1) 31–42 × 9–13 μm, crowded, mostly clavate, slender, often obtuse at apex, sometimes slightly tapered into capitate to sub-capitate, becoming somewhat narrow toward the base; (2) 30–58 × 16–24 μm, occasionally inflated into 73 × 40 μm in size, mostly broadly clavate, balloon-shape, often broadly obtuse to round at apex, then becoming quite narrow at the base; thin- to somewhat thick-walled, hyaline to pale yellow, sometimes with golden yellow to yellowish brown contents. Hymenophoral trama 78–113 μm thick, regular, golden yellow, composed of thin-walled, smooth, cylindric to often inflated hyphae 11–28 μm wide, usually slightly constricted between two connected cells. Veilpellis often with slightly encrusted, hyaline, cylindric hyphae 4–6 μm wide. Pileipellis a cutis, regular to sub-regular, composed of distinct encrusted, thin- to slightly thick-walled, golden yellow to somewhat yellowish brown cylindric hyphae 4–11 μm wide. Pileal trama 19–40 μm wide, hyaline, cylindric, often inflated. Stipitipellis a cutis, regular to sub-regular arranged, sometimes disrupted with entangled, extended hyphae 3–12 μm wide, hyaline, slightly encrusted. Stipe trama hyphae 9–25 μm wide, hyaline to pale yellow. Caulocystidia not observed. Oleiferous hyphae 3–12 μm wide, often observed in all tissues, yellowish brown, usually bent, sometimes diverticulate, occasionally branched. Clamp connections common on all hyphae.

*Habitat*.— gregarious, usually as large, discrete clusters, on clay soil, under *Castanopsis* dominated forest.

*Known distribution*.—**CHINA** (Hainan).

Additional specimens (paratype) examined: **CHINA**. Hainan Province, Wuzhishan, Maoyang Town, Wuzhishan substation of Hainan Tropical Rainforest National Park, August 11, 2021, FYG64412 (FCAS3529), FYG64413 (FCAS3530), and FYG64414 (FCAS3531); Wanning, Changfeng Town, Shanjia Village, May 9, 2020, HN20200509-01 (FCAS3532); Changjiang, Bawangling substation of Hainan Tropical Rainforest National Park, September 2, 2020, Zeng 4940 (FHMU6957).

### Toxin Detection

**Figure 6:**
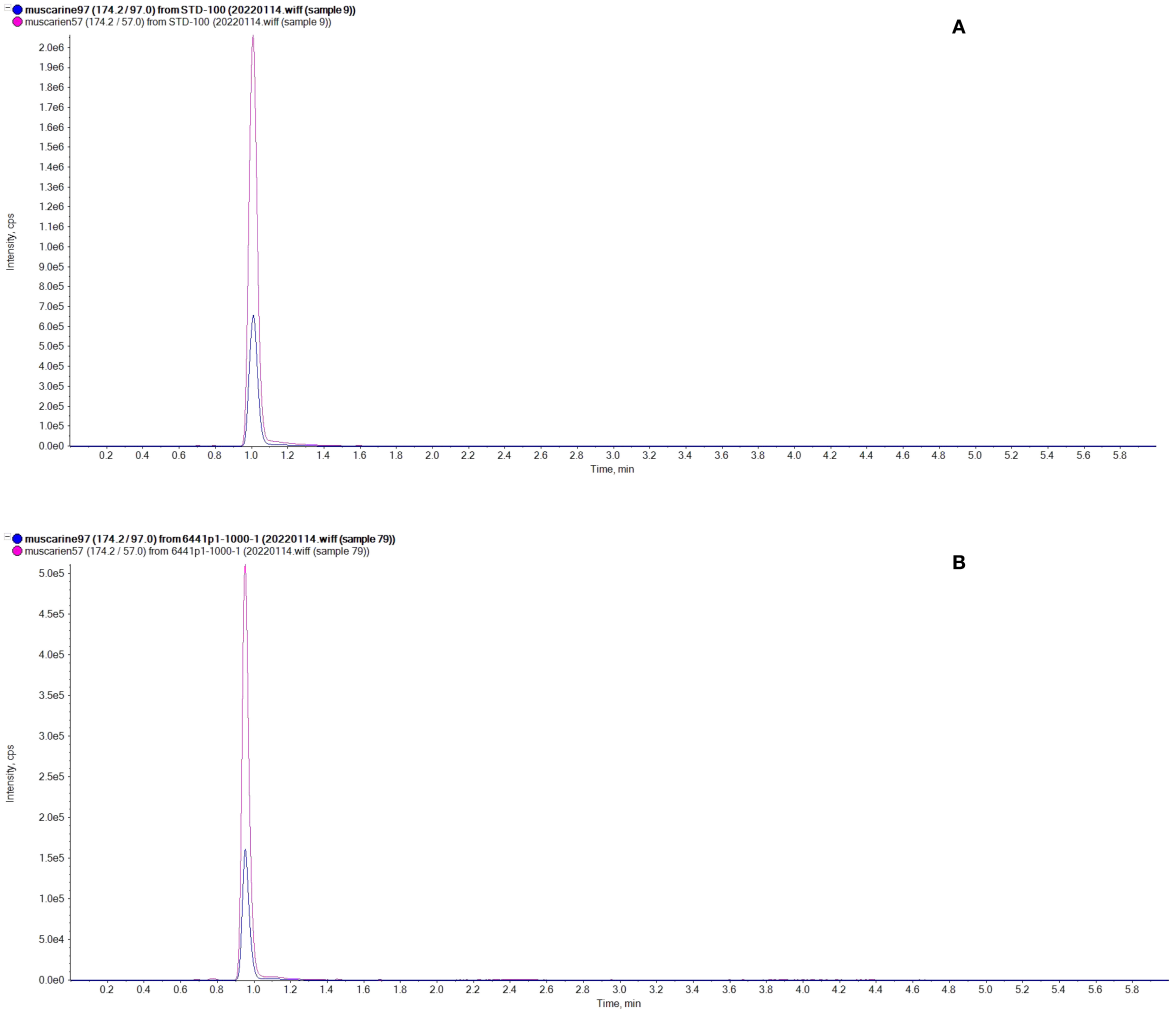
Chromatograms of muscarine. **(A)** Standard representative chromatograms of muscarine (100 ng/mL); **(B)** The representative chromatograms of *Inosperma zonativeliferum*.

In the qualitative analysis, through targeted screening, *Inosperma zonativeliferum* contains muscarine and no other toxins (ibotenic acid, muscimol, psilocybin, psilocin α-amanitin, β-amanitin, γ-amanitin, phalloidin, phallacidin, and phallisacin). In the quantitative analysis, muscarine was detected in the new species by UPLC-MS/MS analysis, and the toxin of muscarine was identified by comparing retention time (0.95 min) and relative deviation (0.32%) in the allowance of ±25% relative range; the chromatograms of muscarine are shown in Figure 6. The calibration curve for muscarine generated during the validation was *y* = 20,223.15,025*x* + 18,054.61816 (*r* = 0.99837) for muscarine concentration in the range of 2–100 ng/mL (*y* means the peak area, and *x* is the muscarine concentration, while *r* means correlation coefficient). The precision was performed, injecting six times the standard mixture, and the relative standard deviation (RSD) was 1.37%. Percentages of recovery are 98.47–100.18%, as well as the average recovery rate was 98.78%. For actual sample analysis, the contents of muscarine in the holotype (FYG6441) were 2.08 ± 0.05 g/kg in the pileus and 6.53 ± 1.88 g/kg in the stipe. For mushrooms that were collected from different regions, the contents of muscarine are 2.08 ± 0.05 g/kg (FYG6441), 0.58 ± 0.02 g/kg (HN20200509-01), and 1.13 ± 0.03 g/kg (Zeng4940) in the pileus, respectively.

## Discussions

The new species was collected in *Castanopsis*-dominated forests mixed with *L. formosana* (Hamamelidaceae), *M. paniculata* (Tiliaceae), *P. rubra* (Rubiaceae), *M. chinensis* (Lauraceae), and *G. oblongifolia* (Clusiaceae) in tropical China. So far, it is known in three localities in Hainan Province in China. In the field, *I. zonativeliferum* can be easily characterized by its gregarious habit, medium-sized basidiomes, distinct whitish veil remnants forming appressed scales on the whole pileus, yellowish white to yellowish brown, and often split pileus with crenate margin. The zonate velar remnants are outstanding in the type materials; they are however not so distinct in certain collections or individuals. The lamellae are often yellowish white to brown, but sometimes pinkish white at a very young age. Microscopically, it can be distinguished by ellipsoid basidiospores, and the shape of cheilocystidia ranged from slenderly clavate to broadly clavate, or sometimes balloon shaped.

In the multi-gene phylogeny, *I. zonativeliferum* was positioned in the Old World tropical clade 2 and was sister to the subclade unifying *Inosperma gregarium* and *I. virosum*, both of which were described from India under Dipterocarpaceae trees. Morphologically, the two Indian species have uniformly brown pileus with indistinct veilpellis, brownish orange stipe, and smaller basidiospores (*I. gregarium*: 7–8.5 × 5–5.5 μm, Q = 1.3–1.8, Q_m_ = 1.6, and *I. virosum*: 6.5–7.5 × 4.5–5.5 μm; Vrinda et al., 1996; Latha and Manimohan, 2016).

In tropical China, three *Inosperma* taxa were recently described, viz. *I. hainanense* Y.G. Fan, L.S. Deng, W.J. Yu & N.K. Zeng, *I. muscarium* Y.G. Fan, L.S. Deng, W.J. Yu & N.K. Zeng (Deng et al., 2021a), and *I. subsphaerosporum* Y.G. Fan, L.S. Deng, W.J. Yu & LY Liu (Deng et al., 2021b). These three species also resemble the new species in their gregarious habit, rather crowded lamellae, and ecology in *Castanopsis* forests. *Inosperma hainanense* and *I. muscarium* differ in having brown to dark brown pileus that lack thick white veilpellis and the lack of any pinkish or orange tinge in lamellae (Deng et al., 2021a). *Inosperma subsphaerosporum* shares similarities in having appressed scaly and yellowish pileus but differs by its subspherical basidiospores and cylindrical clavate cheilocystidia (Deng et al., 2021b).

Additionally, *I. maculatum* (Boud.) Matheny & Esteve-Rav., a species also covered with conspicuous white velar patches in pileus, is similar in having an indistinct or even without umbo on pileus, smooth and subphaseoliform basidiospores measured 8–10.5 × 4.5–6 μm by Kuyper (1986), and slenderly clavate to cylindrico-clavate cheilocystidia (27–66 × 10–25 μm), but it can be distinguished with the new species by its ochraceous brown, pinkish brown, chestnut-brown, date-brown to dark reddish-brown pileus sometimes with purplish or violaceous sheen, bulbous stipe base up to 12 mm wide, strong smell like *Tuber* sp., like *Amanita phalloides* or sometimes smell of raw potatoes, the presence of caulocystidia at stipe apex, and ecology under frondose trees in temperate Europe (Kuyper, 1986; Stangl, 1989; Kropp et al., 2013).

Seven taxa of *Inospema* were reported as muscarine positive, viz. *I. cervicolor* (Pers.) Matheny & Esteve-Rav. (Stijve, 1982), *I. erubescens* (A. Blytt) Matheny & Esteve-Rav. (Stijve, 1982), *I. maculatum* (Boud.) Matheny & Esteve-Rav. (Stijve, 1982; Gurevich and Nezdoiminogo, 1992), *I. vinaceobrunneum* (Matheny, Ovrebo & Kudzma) Haelew. (Kosentka et al., 2013), *I. virosum* (Latha et al., 2020), *I. muscarium*, and *I. hainanense* (Deng et al., 2021a). *Inosperma carnosibulbosum* (CK Pradeep and Matheny) Matheny & Esteve-Rav, a poisonous mushroom causing muscarinic syndromes in India, is probably muscarine positive species (Chandrasekharan et al., 2020; Latha et al., 2020). This study enriched the knowledge of the *Inosperma* diversity and its toxin type and contents, which could provide scientific data for the prevention and treatment of *Inosperma* poisoning.

Muscarine (C_9_H_20_NO${}_{2}^{+}$), is a water-soluble toxin, which can be easily dissolved in water or other solvents with similar polarity to water. High concentrations of muscarine can be usually found in *Inocybaceae* and *Clitocybe* species (Peredy and Bradford, 2014). Brown et al. (1962) found that the contents of muscarine differ in different collections of certain species. Li et al. (2021b) detected two toxic *Inocybe* from China, discovering that the muscarine contents in *Inocybe squarrosolutea* (Corner & E. Horak) Garrido and *Inocybe squarrosofulva* (S.N. Li, Y.G. Fan & ZH Chen) ranged from 136.4 ± 25.4 to 1,683 ± 313 and 31.2 ± 5.8 to 101.8 ± 18.9 mg/kg in dry weight, respectively, showing significant differences among different specimens. In the present study, no amanitin was detected in *I. zonativeliferum*, but the content of muscarine in stipe was three times higher than that in pileus. In addition, muscarine in pileus of *I. zonativeliferum* from the three studied specimens at various locations shows significant differences. The levels of the muscarine collected from Wuzhishan City (FYG6441) were about four times higher than that from Wanning City (HN20200509-01), where the two locations were 124 km away in Hainan province. The differences between muscarine in the pileus and the stipe and different specimens were observed in the present new species; further study is required to elucidate the mechanisms of such differences.

## Data Availability Statement

The datasets presented in this study can be found in online repositories. The names of the repository/repositories and accession number(s) can be found in the article/Supplementary Material.

## Author Contributions

H-JL, FX, and Y-GF: conceptualization, writing—review and editing, supervision, and project administration. L-SD and W-JY: methodology, formal analysis, and writing—original draft preparation. L-SD, W-JY, X-PW, and FX: performing the experiment. Y-GF, H-JL, N-KZ, and L-SD: resources. H-JL, FX, Y-GF, X-PW, and L-SD: funding acquisition. All authors have read and agreed to the published version of the manuscript.

## Funding

This work was supported by the Hainan Basic and Applied Research Project for Cultivating High-Level Talents (No. 2019RC230), the National Natural Science Foundation of China (Grant Nos. 31860009, 31400024, and 31501814), the Natural Science Foundation of Ningxia (No. 2020AAC03437), the Innovative Research Projects for Graduate Students in Hainan Medical University, Hainan China (No. HYYS2020-42), and the Central Public interest Scientific Institution Basal Research Fund (No. 1630082022004).

## Conflict of Interest

The authors declare that the research was conducted in the absence of any commercial or financial relationships that could be construed as a potential conflict of interest.

## Publisher's Note

All claims expressed in this article are solely those of the authors and do not necessarily represent those of their affiliated organizations, or those of the publisher, the editors and the reviewers. Any product that may be evaluated in this article, or claim that may be made by its manufacturer, is not guaranteed or endorsed by the publisher.
