# Supplementary material for: A New Muscarine-Containing Inosperma (Inocybaceae, Agaricales) Species Discovered From One Poisoning Incident Occurring in Tropical China
Source: Front Microbiol. 2022 Jul 4;13:923435. doi: 10.3389/fmicb.2022.923435 (PMC9290438; doi:10.3389/fmicb.2022.923435)
Supplement: Supplementary file 1 [file Data_Sheet_1.PDF]

Supplementary Material (1)

A new, muscarine-containing *Inosperma* (Inocybaceae, Agaricales) species discovered from one poisoning incident occurring in tropical China

Deng Lun-Sha[#], Yu Wen-Jie[#], Zeng Nian-Kai, Zhang Yi-Zhe, Li Hai-Jiao[*], Xu Fei[*], Fan Yu-Guang[*]

[*]**Correspondence:**
Hai-Jiao Li: lihaijiao715@126.com
Fei Xu: lengyue0524@163.com
Fan Yu-Guang: mycena@qq.com
[#]These author contributed equally to this work.

TABLE S1 | Taxon sampling information and DNA sequences used for BLASTn and/or phylogenetic analyses.

Taxa	Collection number/Herbaium	Locality	GenBank accession number			Reference
			ITS	LSU	RPB2	
Inosperma acutofulvum	MCVE29416	Italy	MG944832	–	–	Bizio et al., 2017
Inosperma adaequatum	JV16501F	Finland	–	AY380364	AY333771	Pradeep et al., 2016
Inosperma aff. *lanatodiscum*	PBM3051	USA	JQ801401	JN975026	JQ846485	Pradeep et al., 2016
Inosperma aff. *calamistratum*	DED8134	Thailand	GQ892983	GQ892937		Pradeep et al., 2016
Inosperma aff. *calamistratum*	REH8420	Costa Rica	JQ801390	JN975018	JQ846471	Pradeep et al., 2016
Inosperma aff. *fastigiellum*	PBM3325	USA	JQ801399	JQ815419	JQ846477	Pradeep et al., 2016
Inosperma aff. *latericium*	TR109-02	Papua New Guinea	JQ801405	JN975023	JQ846487	Pradeep et al., 2016
Inosperma africanum	HLA0353	Benin	MT534299	–	–	Aïgnon et al., 2021
Inosperma akirnum	CAL1358	India	KY440085	KY549115	KY553236	Latha and Manimohan, 2016
Inosperma apiosmotum	PBM3020	USA	JQ801385	JN975021	JQ846463	Pradeep et al., 2016

Inosperma bicoloratum	ZT12187	Malaysia	GQ892984	GQ892938	JQ846464	Pradeep et al., 2016
Inosperma bongardii	JV7450F	Finland	—	EU555448	—	Pradeep et al., 2016
Inosperma bulbomarginatum	MR00357	Benin	MN096190	MN097882	MN200775	Aïgnon et al., 2021
Inosperma bulbomarginatum	PC96082	Benin	JQ801412	JN975027	—	Aïgnon et al., 2021
Inosperma calamistratoides	PBM3384	Australia	JQ801393	JQ815415	KJ729949	Pradeep et al., 2016
Inosperma calamistratum	PBM1105	USA	JQ801386	JQ815409	JQ846466	Pradeep et al., 2016
Inosperma calamistratum	EL1904	Sweden	AM882938	AM882938	—	Pradeep et al., 2016
Inosperma calamistratum	PBM2351	USA	—	AY380368	AY333764	Pradeep et al., 2016
Inosperma calamistratum	JV11950	Latvia	—	EU555452	AY333763	Pradeep et al., 2016
Inosperma calamistratum	TR74-06	Papua New Guinea	JQ801391	JN975020	JQ846472	Pradeep et al., 2016
Inosperma carnosibulbosum	TBGT12047	India	KT329448	KT329454	KT329443	Pradeep et al., 2016
Inosperma cervicolor	TURA4761	Finland	JQ801395	JQ815417	JQ846474	Pradeep et al., 2016
Inosperma cf. *lanatodiscum*	TURA1812	Finland	JQ408763	JQ319694	JQ846484	Pradeep et al., 2016
Inosperma cf. *reisneri*	MCA646	Japan	—	EU555463	—	Pradeep et al., 2016

Inosperma changbaiense	FYG2010156 (Type)	China	MH047251	MG844976	MT086755	Bau and Fan, 2018
Inosperma cyanotrichium	I37	Australia	JQ801396	JN975033	JQ846476	Pradeep et al., 2016
Inosperma dodonae	STU:SMNS-STU-F-0901253	Netherlands	MW647615	—	—	Bandini et al., 2021
Inosperma erubescens	JV9070F	Finland	—	EU569846	—	Pradeep et al., 2016
Inosperma flavobrunneum	HLA0372	Benin	MT534290	MT536756	—	Aïgnon et al., 2021
Inosperma flavobrunneum	HLA0367	Benin	MN096199	MT536754	—	Aïgnon et al., 2021
Inosperma geraniodorum	EL10606	Sweden	FN550945	FN550945	—	Pradeep et al., 2016
Inosperma gregarium	ZT8944	India	—	EU600903	EU600902	Pradeep et al., 2016
Inosperma gregarium	CAL1309	India	KX852305	KX852306	KX852307	Latha and Manimohan, 2016
Inosperma hainanense	Zeng4937 (holotype)	China	MZ374070	MZ374761	MZ388104	Deng et al., 2021a
Inosperma ismeneanum	STU:SMNS-STU-F-0901561	Germany	MW647625	—	—	Bandini et al, 2021
Inosperma lanatodiscum	PBM2451	USA	JQ408759	JQ319690	JQ846483	Pradeep et al., 2016

Inosperma latericium	PDD92382	New Zealand	GU233367	GU233413	—	Pradeep et al., 2016
Inosperma maculatum	EL12604	Sweden	AM882964	AM882964	—	Pradeep et al., 2016
Inosperma maculatum	PBM2446	USA	DQ241778	AY745700	EU569863	Pradeep et al., 2016
Inosperma maximum	PBM2222	USA	—	EU569854	—	Pradeep et al., 2016
Inosperma misakaense	PC96234	Zambia	JQ801409	EU569875	AY333767	Pradeep et al., 2016
Inosperma monastichum	STU:SMNS-STU-F-0901533	Germany	MW647631	—	—	Bandini et al., 2021
Inosperma mucidiolens	DG1824 (Type)	Canada	HQ201339	HQ201340	—	Pradeep et al., 2016
Inosperma muscarium	FYG6091 (holotype)	China	MZ373982	MZ373991	MZ388093	Deng et al., 2021a
Inosperma mutatum	PBM2542	USA	—	AY732212	DQ472729	Pradeep et al., 2016
Inosperma neobrunnescens	PBM2452	USA	—	EU569868	EU569867	Pradeep et al., 2016
Inosperma neobrunnescens var. *leucothelotum*	SAT0427406	USA	JQ801411	JN975025	JQ846489	Pradeep et al., 2016
Inosperma proximum	ZT13015	Thailand	EU600839	EU600840	—	Pradeep et al., 2016

Inosperma quietiodor	EL11504	Sweden	AM882960	AM882960	—	Pradeep et al., 2016
Inosperma bulbomarginatum	PC96082	Zambia	JQ801412	JN975027	—	Aïgnon et al., 2021
Inosperma rhodiolum	EL223-06	France	FJ904175	FJ904175	—	Pradeep et al., 2016
Inosperma rimosoides	PBM2459	USA	DQ404391	AY702014	DQ385884	Pradeep et al., 2016
Inosperma rubricosum	PBM3784	Australia	KP308817	KP170990	KM406230	Pradeep et al., 2016
Inosperma saragum	CAL1360	India	KY440103	KY549133	KY553249	Pradeep et al., 2016
Inosperma shawarense	ASSE79	Pakistan	KY616964	KY616966	—	Naseer, et al., 2018
Inosperma sp.	PC96013	Zambia	JQ801383	EU600883	EU600882	Pradeep et al., 2016
Inosperma sp.	PBM2871	USA	HQ201348	HQ201348	JQ846475	Pradeep et al., 2016
Inosperma sp.	BB3233	Zambia	JQ801415	EU600885	—	Pradeep et al., 2016
Inosperma sp.	L-GN3a	Papua New Guinea	JX316732	JX316732	—	Pradeep et al., 2016
Inosperma sp.	TJB10045	Thailand	KT600658	KT600659	KT600660	Pradeep et al., 2016
Inosperma sp.	TR22006	Papua New Guinea	JQ801416	JN975017	JQ846496	Pradeep et al., 2016

Inosperma subhirsutum	PC96073	Zambia	JQ801417	EU600870	EU600869	Pradeep et al., 2016
Inosperma subsphaerosproum	FYG5848	China	MW403825	MW397171	MW404237	Deng, et al., 2021b
Inosperma vinaceobrunneum	PBM2951	USA	—	HQ201353	JQ846478	Pradeep et al., 2016
Inosperma vinaceum	AMB18747	Italy	MW561108	MW561120	—	Cervini, et al., 2021
Inosperma viridipes	I153	Australia	KP641646	KP171095	KM656139	Pradeep et al., 2016
Inosperma virosum	TBGT753	India	KT329452	KT329458	KT329446	Pradeep et al., 2016
Inosperma virosum	CAL1383	India	KY440108	KY549138	KY553253	Vrinda et al., 1996
Inosperma zonativeliferum	**FYG6441 (holotype)**	**China**	**OL850878**	**OM845772**	**ON075044**	**The present study**
Inosperma zonativeliferum	**FYG64412**	**China**	**OM845769**	**OM845773**	**ON086764**	**The present study**
Inosperma zonativeliferum	**FYG64413**	**China**	**OM845770**	**OM845774**	**ON152702**	**The present study**
Inosperma zonativeliferum	**FYG64414**	**China**	**OM845771**	**OM845775**	—	**The present study**
Inosperma zonativeliferum	**Zeng4940**	**China**	**ON182026**	**ON182027**	**ON182027**	**The present study**
Inosperma zonativeliferum	**HN20200509-01**	**China**	**ON182025**	—	—	**The present study**

References

Aïgnon, H.L., Jabeen, S., Naseer, A., Yorou, N.S., and Ryberg, M. (2021). Three new species of *Inosperma* (Agaricales, Inocybaceae) from Tropical Africa. *MycoKeys* 77, 97–116. doi: 10.3897/mycokeys.77.60084

Bandini, D., Oertel, B., and Eberhardt, U. (2021). Even more fibre-caps (2): Thirteen new species of the family Inocybaceae. *Mycologia Bavarica* 21, 27–98.

Bau, T., and Fan, Y.G. (2018). Three new species of *Inocybe* sect. *Rimosae* from China. *Mycosystema* 37, 693–702. doi: 10.13346/j.mycosystema.180033

Bizio E., and Castellan A., (2017) *Inocybe acutofulva* and *Inocybe grammopodia* var. *paleoveneta*, two news taxa from Alta Marca Trevigiana (Treviso, Veneto, Italy).

Cervini, M., Carbone, M., and Bizio, E. (2021). *Inosperma vinaceum*, una nuova specie distinta da *I. rhodiolum* e *I. adaequatum*. *Rivista di Micologia* 63, 215–241.

Deng, L.S., Kang, R., Zeng, N.K., Yu, W.J., Chang, C., Xu, F., Deng, W.Q., Qi, L.L., Zhou, Y.L., and Fan, Y.G. (2021a). Two new *Inosperma* (Inocybaceae) species with unexpected muscarine contents from tropical China. *MycoKeys* 85, 87–108. doi: 10.3897/mycokeys.85.71957

Deng, L.S., Yu, W.J., Zeng, N.K., Liu, L.J., Liu, L.Y., and Fan, Y.G. (2021b). *Inosperma subsphaerosporum* (Inocybaceae), a new species from Hainan, tropical China. *Phytotaxa* 502, 169–178. doi: 10.11646/phytotaxa.502.2.5

Latha, K.P.D., and Manimohan, P. (2016). *Inocybe gregaria*, a new species of the *Inosperma* clade from tropical India. *Phytotaxa* 286, 107–115. doi: 10.11646/phytotaxa.286.2.5

Naseer, A., Khalid, A.N., and Smith, M.E. (2017). *Inocybe shawarensis* sp. nov. in the *Inosperma* clade from Pakistan. *Mycotaxon -Ithaca Ny-* 132, 909–918. doi: 10.5248/132.909

Pradeep, C.K., Vrinda, K.B., Varghese, S.P., Korotkin, H.B., and Matheny, P.B. (2016). New and noteworthy species of *Inocybe* (Agaricales) from tropical India. *Mycological Progress* 15(3): 1–25. doi: 10.1007/s11557-016-1174-z.

Vrinda, B., Pradeep, C., Joseph, A., and Abraham, T.K. (1996). A new *Inocybe* (Cortinariaceae) from Kerala state, India. *Mycotaxon* 57, 171–174.
