# Supplementary material for: A New Muscarine-Containing Inosperma (Inocybaceae, Agaricales) Species Discovered From One Poisoning Incident Occurring in Tropical China
Source: Front Microbiol. 2022 Jul 4;13:923435. doi: 10.3389/fmicb.2022.923435 (PMC9290438; doi:10.3389/fmicb.2022.923435)
Supplement: Supplementary file 2 [file Data_Sheet_2.PDF]

Supplementary Material (2)

A new, muscarine-containing *Inosperma* (Inocybaceae, Agaricales) species discovered from one poisoning incident occurring in tropical China

Deng Lun-Sha[#], Yu Wen-Jie[#], Zeng Nian-Kai, Zhang Yi-Zhe, Li Hai-Jiao[*], Xu Fei[*], Fan Yu-Guang[*]

[*]**Correspondence:**
Hai-Jiao Li: lihaijiao715@126.com
Fei Xu: lengyue0524@163.com
Fan Yu-Guang: mycena@qq.com
[#]These author contributed equally to this work.

Supplementary Material (2): The original data of UPLC-MS/MS in manuscript of "A new, muscarine-containing *Inosperma* (Inocybaceae, Agaricales) species discovered from one poisoning incident occurring in tropical China".

(1) The linear range

Analyte Name: muscarine97
Internal Standard: N/A

Data File	20220114.wiff	Result Table	FYG20220114
Acquisition Date	1/14/2022 10:31:00 AM	Algorithm Used	MQ4
Acquisition Method	mushroom 174.2 to 97 57.dam	Instrument Name	Triple Quad 6500+ Low Mass
Project	N/A		

Regression Equation: $y = 20223.15025\,x + 18054.61816$ ($r = 0.99837$, $r^2 = 0.99675$) (weighting: None)

Expected Concentration	Number of Values	Mean Calculated Concentration (nM/L)	% Accuracy	Std. Deviation	%CV
2.00	3 of 3	1.870	93.5	0.06	3.1
6.25	3 of 3	6.762	108.2	0.56	8.2

12.50	3 of 3	12.908	103.3	1.00	7.8
25.00	3 of 3	23.414	93.7	2.41	10.3
50.00	3 of 3	50.960	101.9	1.78	3.5
100.00	3 of 3	99.836	99.8	4.22	4.2

Analyte Name: muscarine97

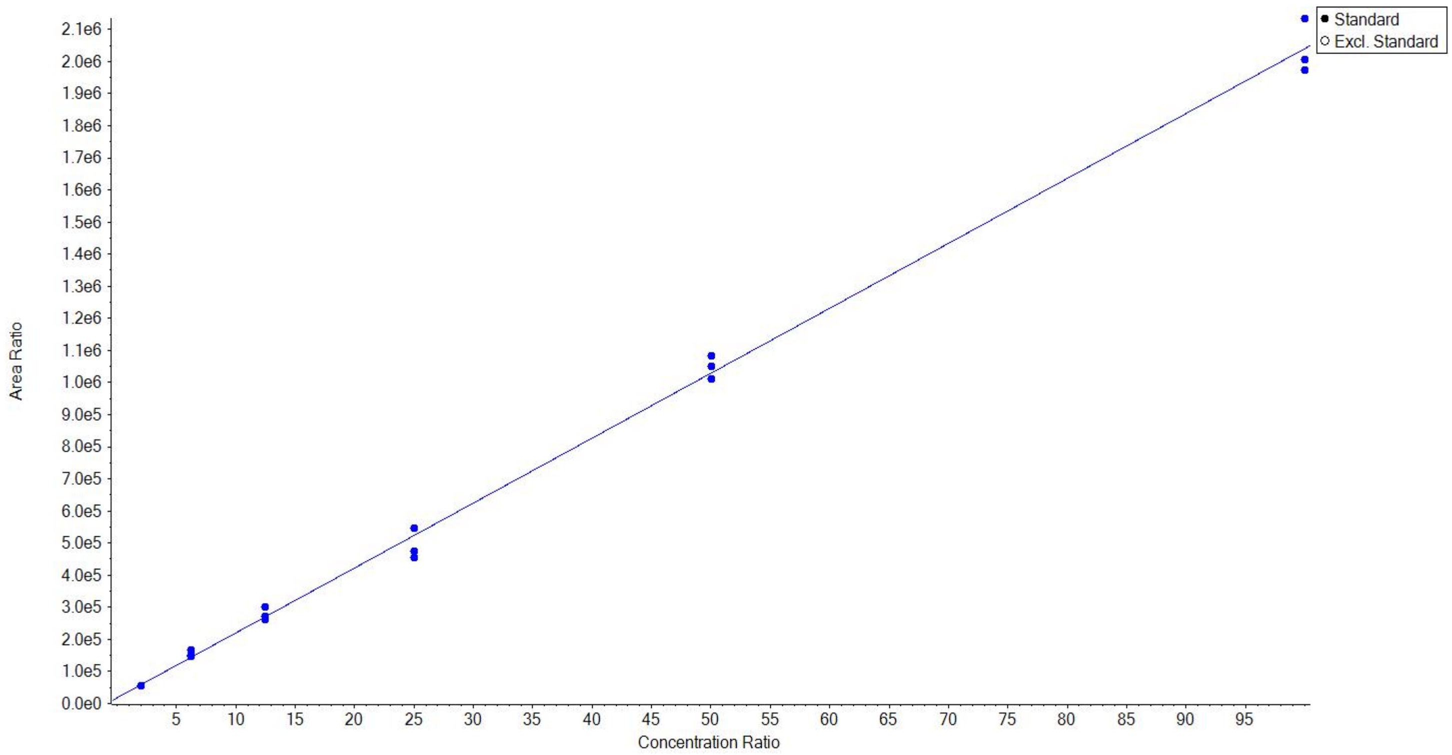

(2) The recovery data. (Table 2-1)

Table 2-1.

Name	Also known as	weight (g)	ng/mL	Extracted volume (mL)	Final volume (mL)	ng	Minus standard	RSD	Average RSD
37-bendi-1-1000-1	Sample value	0.0209	5.3320	1.782	2	10.664			
37-bendi-1-1000-2	Sample value	0.0209	5.5900	1.782	2	11.18			
37-bendi-2-1000-1	Sample value	0.0203	4.4940	1.782	2	8.988			
37-bendi-2-1000-2	Sample value	0.0203	4.4600	1.782	2	8.92	10.061357		
37-jiabiao-1-1000-1	Standard value	0.0208	5.9230	1.782	2	10.5548	0.005008	100.1835	

37-jiabiao-1-1000-2	Standard value	0.0208	5.9210	1.782	2	10.5512	0.004934	98.7006	
37-jiabiao-2-1000-1	Standard value	0.0200	5.9250	1.782	2	10.5584	0.004887	97.7561	
37-jiabiao-2-1000-2	Standard value	0.0200	5.9260	1.782	2	10.5601	0.004923	98.4690	98.7773

(3) The precision data. (Table 2-2)

Table 2-2.

Name	Standard concentration (ng/ml)	ng/ml
STD12.5-Precision-1	12.5	12.05
STD12.5-Precision-2	12.5	11.84
STD12.5-Precision-3	12.5	11.69
STD12.5-Precision-4	12.5	11.656
STD12.5-Precision-5	12.5	11.684

Name	Standard concentration (ng/ml)	ng/ml
STD12.5-Precision-6	12.5	11.617
STDEV		0.16
AVERAGE		11.75
RSD		1.37

(4) Sample data. (Table 2-3 and 2-4)

Table 2-3.

Sample Name	Sample Type	Area (cps)	RT (min)	Target [Conc]. (nM/L)	Calculated Conc.(nM/L)
blank	Unknown	214.1	0.81	N/A	< 0
6441p1-1000-1	Unknown	429200	0.95	N/A	20.033
6441p1-1000-2	Unknown	446700	0.95	N/A	20.878
6441p2-1000-1	Unknown	453000	0.95	N/A	21.183
6441p2-1000-2	Unknown	459300	0.95	N/A	21.491

blank	Unknown	297.8	0.94	N/A	< 0
6441s1-1000-1	Unknown	1005000	0.95	N/A	47.93
6441s1-1000-2	Unknown	995800	0.95	N/A	47.469
6441s2-1000-1	Unknown	1693000	0.95	N/A	81.212
6441s2-1000-2	Unknown	1694000	0.95	N/A	81.263

Table 2-4.

Name	ng/ml	weight (g)	mg/kg	Average value (mg/kg)	Average value (g/kg)	STDEV (mg/kg)	STDEV (g/kg)
6441s1-1000-1	48.818	0.0198	4931.1111				
6441s1-1000-2	48.348	0.0198	4883.6364				
6441s2-1000-1	82.806	0.0203	8158.2266				

Name	ng/ml	weight (g)	mg/kg	Average value (mg/kg)	Average value (g/kg)	STDEV (mg/kg)	STDEV (g/kg)
6441s2-1000-2	82.857	0.0203	8163.2512	6534.0563	6.53	1878.4324	1.88
6441p1-1000-1	20.331	0.0197	2064.0609		0.00		0.00
6441p1-1000-2	21.194	0.0197	2151.6751		0.00		0.00
6441p2-1000-1	21.506	0.0212	2028.8679		0.00		0.00
6441p2-1000-2	21.82	0.0212	2058.4906	2075.7736	2.08	52.9058	0.05

(5) The chromatograms of samples were shown as below.

6441p1-1000-1

RT (Exp. RT):	0.95 (0.95) min
Calculated Conc:	20.033 nM/L
Area:	4.292e5
Sample Type:	(Unknown)

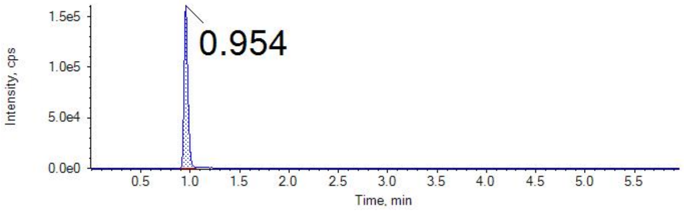

6441p1-1000-2

RT (Exp. RT):	0.95 (0.95) min
Calculated Conc:	20.878 nM/L
Area:	4.467e5
Sample Type:	(Unknown)

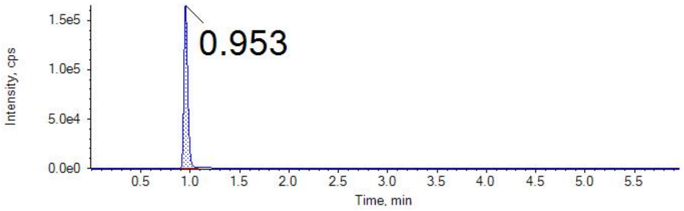

6441p2-1000-1<br><br>RT (Exp. RT): 0.95 (0.95) min<br><br>Calculated Conc: 21.183 nM/L<br><br>Area: 4.530e5<br><br>Sample Type: (Unknown)	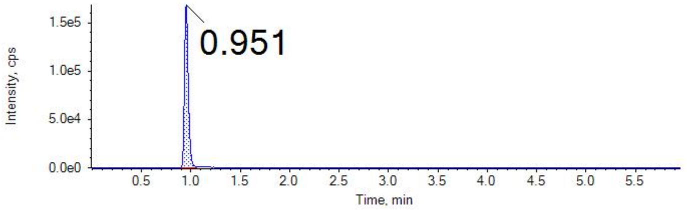

6441p2-1000-2

RT (Exp. RT):	0.95 (0.95) min
Calculated Conc:	21.491 nM/L
Area:	4.593e5
Sample Type:	(Unknown)

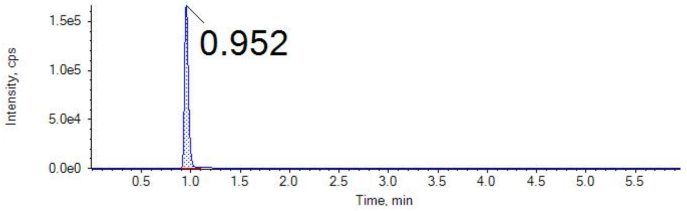

6441s1-1000-1

RT (Exp. RT):	0.95 (0.95) min
Calculated Conc:	47.930 nM/L
Area:	1.005e6
Sample Type:	(Unknown)

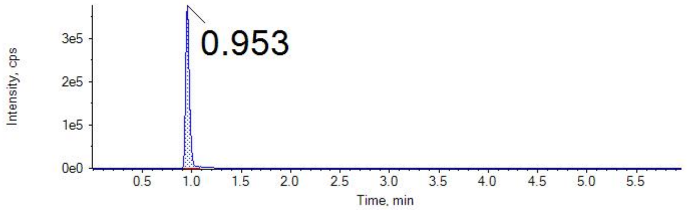

6441s1-1000-2		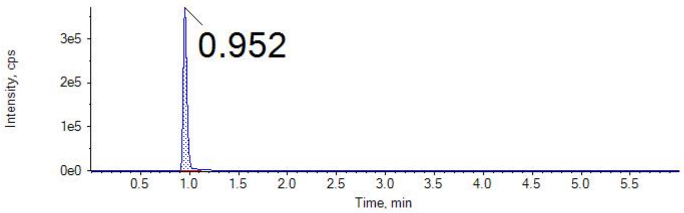
RT (Exp. RT):	0.95 (1.04) min	
Calculated Conc:	47.469 nM/L	
Area:	9.958e5	
Sample Type:	(Unknown)	

6441s2-1000-1

RT (Exp. RT):	0.95 (0.95) min
Calculated Conc:	81.212 nM/L
Area:	1.693e6
Sample Type:	(Unknown)

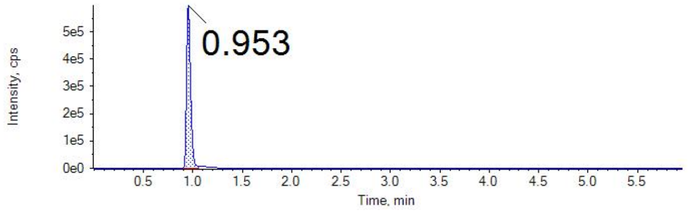

6441s2-1000-2

RT (Exp. RT):	0.95 (1.04) min
Calculated Conc:	81.263 nM/L
Area:	1.694e6
Sample Type:	(Unknown)

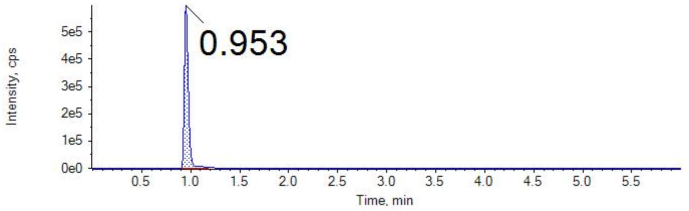

Abbreviations: p = pileus; s = stipe; STD = Standard; STDEV = Standard deviation; RSD = Relative standard deviation; RT = Retention time.
